# Analysis of the Role of Vasa Vasorum in the Oxygen Transport to the Aneurysm Wall

**DOI:** 10.1002/cnm.70051

**Published:** 2025-06-10

**Authors:** Juan R. Cebral, Fernando Mut, Rainald Lohner, Mukhayyirkhuja Abdurakhmonov, Mehdi Ramezanpour, Yasutake Tobe, Anne M. Robertson

**Affiliations:** ^1^ Bioengineering Department George Mason University Fairfax Virginia USA; ^2^ Physics Department George Mason University Fairfax Virginia USA; ^3^ Department of Mechanical Engineering and Materials Science University of Pittsburgh Pittsburgh Pennsylvania USA

**Keywords:** cerebral aneurysm, computational modeling, oxygen transport, vasa vasorum

## Abstract

The role of the vasa vasorum in the growth and rupture of intracranial aneurysms, as well as the conditions stimulating its local development along aneurysm walls are not completely clear and have not been studied on an aneurysm‐specific basis. In this study, the oxygen distribution throughout the wall of an intracranial aneurysm that underwent substantial thickening and developed an extensive adventitial vasa vasorum network was numerically modeled in order to elucidate the role played by the vasa vasorum. The computational model was constructed based on high‐resolution ex vivo micro computed tomography and multi‐photon microscopy images of a tissue sample of the aneurysm harvested during open surgery. The mathematical model was based on the transport equation including oxygen diffusion and consumption in the tissue and diffusion across the lumen in the intimal side, and the vasa vasorum in the adventitial side. The governing equation was numerically solved with a finite volume approach on a high‐resolution mesh containing approximately 48 million tetrahedra with an element size of 10 μm. The results demonstrate that the observed vasa vasorum plexus provided adequate oxygen supply to the outer layers of the thickened walls. Furthermore, the models show that without the vasa vasorum, due to consumption throughout the wall, the oxygen demand could not be met by diffusion from the luminal surface. These findings support the idea that local hypoxic conditions in regions of increased wall thickness stimulate the development of the vasa vasorum network on the adventitial surface.

## Graphical abstract

The observed vasa vasorum network provided adequate oxygen supply to the outer layers of the thickened aneurysm walls, but without the vasa‐vasorum, due to consumption throughout the wall, the oxygen demand could not be met by diffusion from the luminal surface alone. These findings support the idea that local hypoxic conditions in regions of increased wall thickness stimulate the development of the vasa‐vasorum network on the adventitial surface.

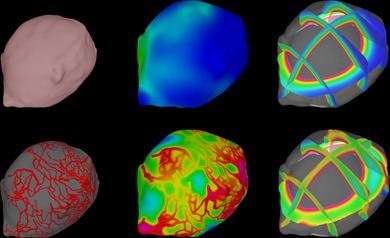

## 1 Introduction

The growth of intracranial aneurysms (IAs) has been advocated as an important marker for imminent rupture [1]. Nevertheless, the multi‐factorial mechanisms responsible for aneurysm growth and ultimately rupture are not completely understood [2]. It is generally accepted that the mechanisms leading to aneurysm enlargement are interrelated to processes of remodeling and structural changes of the wall induced by adverse hemodynamic conditions [3]. In particular, low flow environments have been associated with wall thickening, atherosclerotic alteration, and aneurysm growth [4, 5]. However, as the aneurysm wall thickens during its progression, the amount of oxygen that diffuses from the luminal blood and reaches the outer layers of the wall (the adventitia) may become insufficient to meet the metabolic demands of the cells in the wall [6]. As such, continued aneurysm growth necessitates that this metabolic demand be satisfied.

The vasa vasorum, a system of micro‐vessels permeating the vascular wall, has recently received increased attention as a key player in aneurysm enlargement and inflammation [7, 8]. The vasa vasorum is common in large arteries such as the aorta, but very rare in healthy intracranial vessels [8, 9]. In intracranial arteries, small increases in metabolic demands are thought to be supplied by the cerebrospinal fluid (CSF) surrounding the brain vessels [10], which typically contain low oxygen concentrations [11]. On the other hand, histological examinations of aneurysm tissue have shown that vasa vasorum is quite common in IAs (vasa vasorum was observed in over half of the samples) [8]. Furthermore, the vasa vasorum has been mainly observed on the adventitia side of IA walls (i.e., so‐called vasa vasorum externa) [7]. Although some studies have detected vasa vasorum in large or giant IAs, others have shown that the presence of vasa vasorum was independent of aneurysm size [8, 9].

The vasa vasorum is thought to develop and proliferate primarily in response to expression of hypoxia factors that stimulate angiogenesis and neovascularization [6, 12]. Early studies of aortas argued that the effective oxygen diffusion distance was smaller than the wall thickness, creating an unmet metabolic demand in the outer wall that results in hypoxic conditions that stimulates the formation of the vasa vasorum [6, 13]. Additionally, vasa vasorum development may be induced by stimuli other than vessel wall thickness such as inflammation and atherosclerosis [9, 14]. For instance, in the chronic inflammatory environment of atherosclerosis factors like TNFα and VEGF and others that promote inflammation also stimulate neovascularization and expansion of the vasa vasorum [15]. Once formed, the vasa vasorum can become an alternative avenue for inflammatory cells to further infiltrate the vascular wall [7]. Wall inflammation and the release of growth factors can lead to mural cell proliferation (and associated increased oxygen demand) and wall weakening, thus favoring further aneurysm growth [6, 12].

The purpose of the current study was to investigate the oxygen distribution throughout the wall of an IA that underwent substantial thickening and developed an extensive vasa vasorum network on the adventitial layer in order to elucidate the role played by the vasa vasorum. To this end, a computational model of oxygen transport was developed based on high‐resolution multi‐modality ex vivo imaging of a tissue sample of the aneurysm harvested during open surgery.

## 2 Methods

### 2.1 Data

Data from a 45‐year‐old male patient with multiple IAs who underwent surgical clipping was analyzed. The patient had an 8 mm ruptured aneurysm in the anterior communicating artery (ACOM), a second 4 mm unruptured aneurysm in the M1 segment of the middle cerebral artery (MCA), and a third small 3 mm unruptured aneurysm in the cavernous segment of the internal carotid artery (ICA).

The data collected included: (a) pre‐surgical 3D rotational angiography (3DRA) image, (b) surgical video obtained during open surgery clipping of the MCA aneurysm, (c) tissue sample from the MCA aneurysm, and (d) patient demographic and clinical information.

The protocol for patient consent, handling of patient data, tissue harvest, and analysis was approved by the institutional review boards at Allegheny General Hospital, the University of Pittsburgh, and George Mason University.

### 2.2 Vascular Model

**FIGURE 1:**
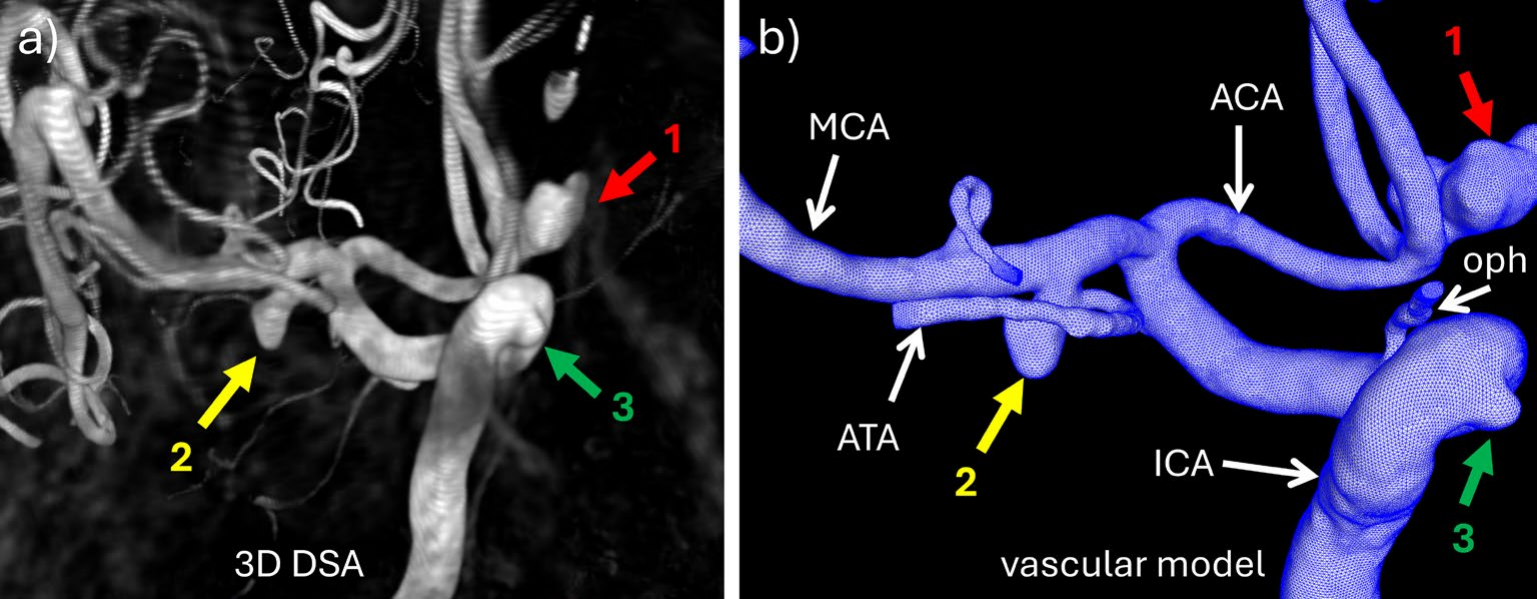
(a) Pre‐surgical 3DRA image of patient with three aneurysms (1 = ACOM, 2 = M1‐MCA, 3 = ICA cavernous), (b) reconstructed vascular model including the three aneurysms. ACA = anterior cerebral artery, ACOM = anterior communicating artery, ATA = anterior temporal artery, ICA = internal carotid artery, MCA = middle cerebral artery, oph = ophthalmic artery.

As shown in Figure 1, a patient‐specific vascular model was reconstructed from the pre‐surgical 3DRA image acquired during contrast injection in the right ICA. Previously described methods were used for image segmentation, surface smoothing, vessel truncation, and mesh optimization [16]. The model contained all three aneurysms (1 = ACOM‐red arrow, 2 = MCA‐yellow arrow, 3 = ICA‐green arrow) and included the ICA, MCA, and anterior cerebral arteries (ACA), as well as smaller branches such as the anterior temporal artery (ATA) emanating from the MCA aneurysm neck, the ophthalmic artery, and the M2 segments of the MCA.

**FIGURE 2:**
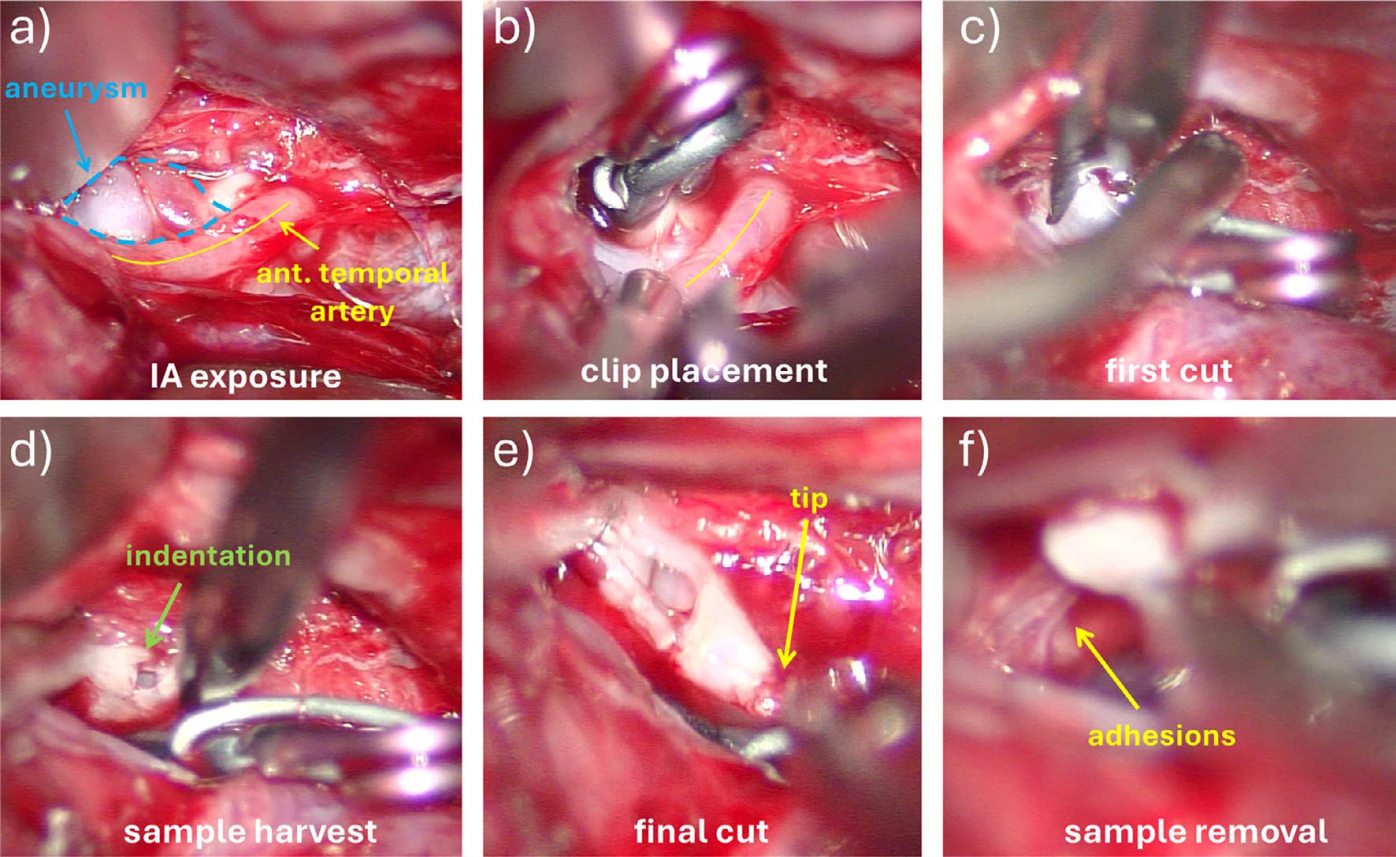
Tissue harvesting from MCA aneurysm: (a) aneurysm exposure, (b) clip placement leaving the anterior temporal branch open, (c) initial cutting of the tissue sample, (d) indentation observed in tissue sample during harvesting, (e) final cut liberating the tissue sample, and (f) adhesions between harvested sample and surrounding tissue observed during sample removal.

During open surgery, after securing the MCA aneurysm by clip placement, a tissue sample was resected from the aneurysm dome. Figure 2 shows a series of frames that illustrate the exposure of the aneurysm, the placement of the clip across the aneurysm neck while avoiding the ATA origin, the initial cut of the aneurysm sample which left a small dent or indentation on one side of the sample, and the final cut that left a small “tip” on the opposite side of the sample. These features were later used to understand the orientation of the harvested sample with respect to the 3D vascular model of the aneurysm.

### 2.3 Hemodynamics Model

The blood flow in the aneurysm and the connected vessels was modeled by solving the unsteady Navier–Stokes equations using an implicit finite elements approach [17] on unstructured grids composed of tetrahedral elements generated by the advancing front method [18, 19]. Pulsatile flow conditions were prescribed at the inlet of the ICA using the Womersley velocity profile [20], and flow rates derived from empirical population averaged flow conditions scaled with the inlet diameter [21, 22]. Outflow boundary conditions consistent with the principle of minimum work (Murray's law) were prescribed at the model outlets. Namely, pressure‐based boundary conditions were applied at the M2 outlets, while flow‐based boundary conditions were prescribed at the outlets the other smaller branches according to the expected flow division given by Murray's law. Walls were approximated as rigid and blood as a Newtonian incompressible fluid. Minimum mesh resolution (i.e., maximum element size) was 0.2 mm and at least 20 nodes across vessel cross‐sections, and simulations were run for two cardiac cycles with 100 timesteps per cycle. This choice of model parameters and approximations has been previously shown to yield reasonable hemodynamic results [23].

### 2.4 Tissue Model

**FIGURE 3:**
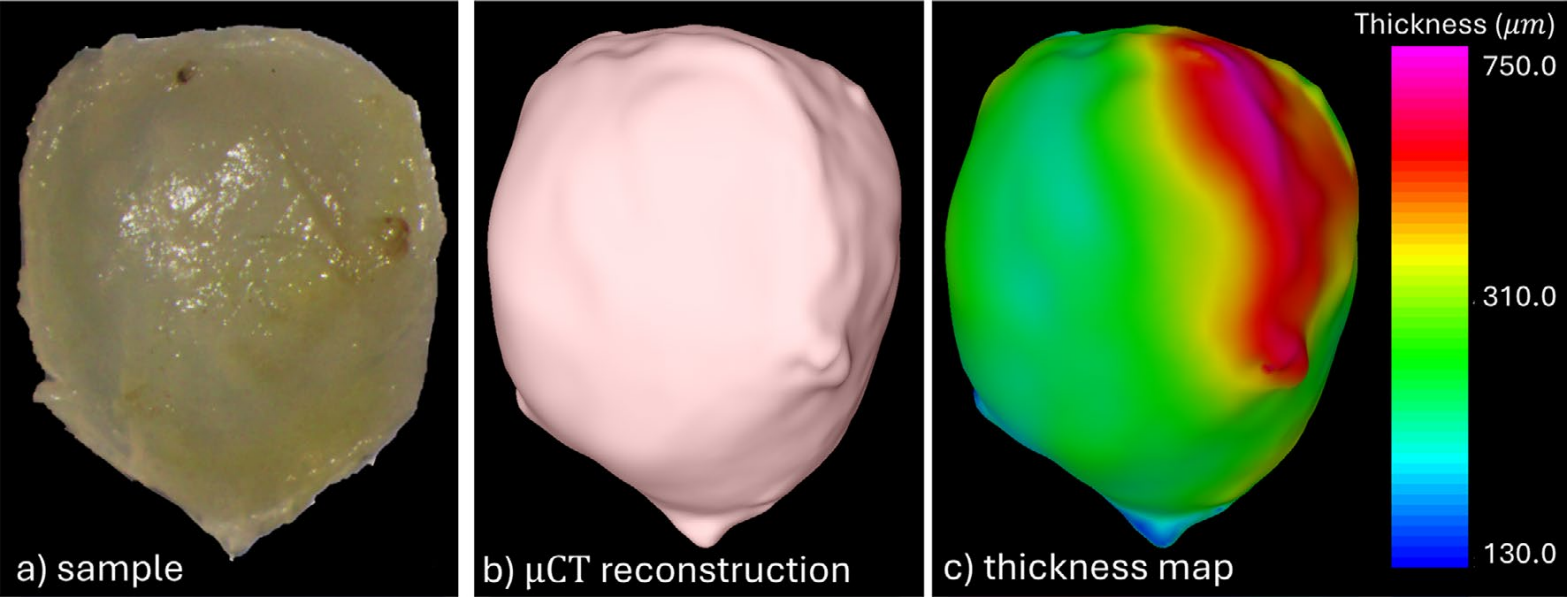
(a) gross anatomical view of resected tissue sample [26], (b) tissue model reconstructed from μCT scan, (c) thickness map computed on reconstructed tissue model.

As in previous studies [23, 24], immediately after harvesting, the resected MCA aneurysm tissue sample was transported to the University of Pittsburgh in a vial with HypoThermosol within an insulated cooler and then fixed in 4% paraformaldehyde within 24 h. Subsequently, the specimen was imaged ex vivo with micro‐CT (μCT) at an imaging resolution of approximately 3 μm. A 3D model (surface triangulation) of the resected sample was then constructed by segmentation of the using Mimics (Materialize GmbH, Munich, Germany) [25]. Since the specimen was slightly bent when placing it in the vial for μCT imaging, the reconstructed geometry was subsequently “corrected” using a finite element elastic deformation to match the original surface imaged with multi‐photon microscopy [25]. Next, local tissue thickness was quantified by computing the distance map between the luminal and abluminal surfaces of the tissue model using 3Matic (Materialize GmbH, Munich, Germany), a software tool for cleaning up surface meshes. Finally, a 3D unstructured mesh composed of tetrahedral elements was generated within the volume of the tissue model using and advancing front method. A uniform element size of 10 μm was prescribed resulting in a mesh of approximately 48 million elements. Figure 3 shows a scope photo of the resected tissue sample (a) [26], the 3D model reconstructed from the μCT scan of the sample (b), and the local wall thickness map (c).

### 2.5 Vasa Vasorum Model

**FIGURE 4:**
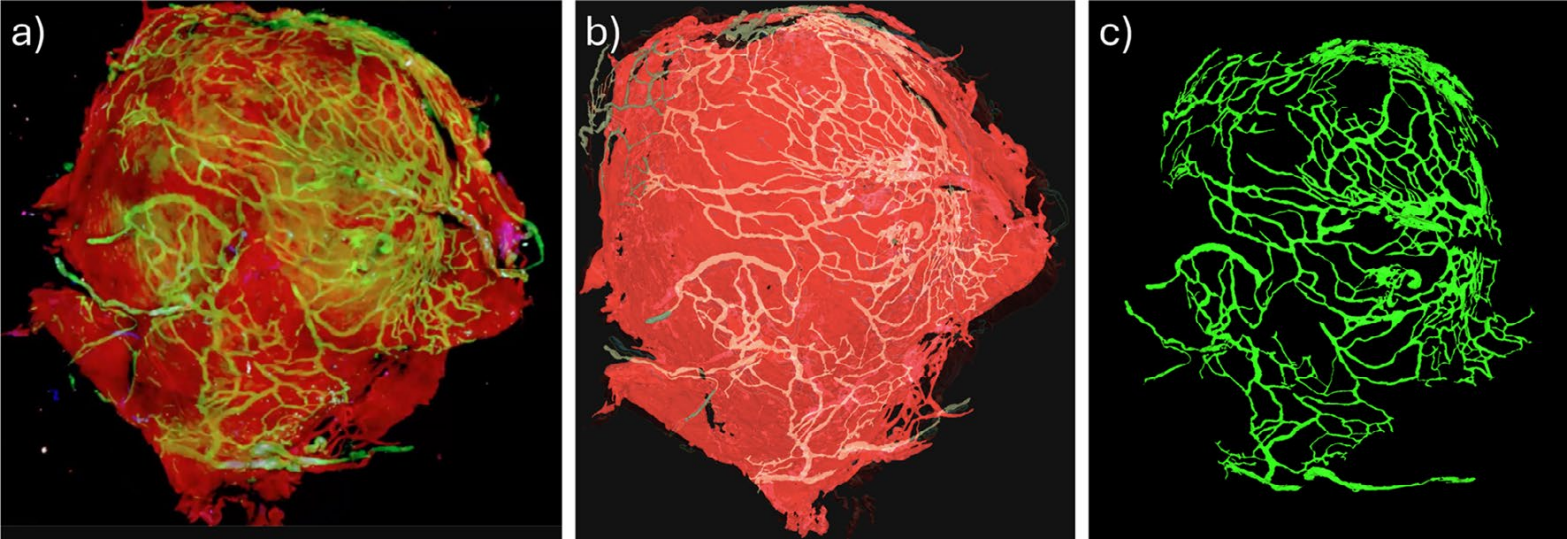
Multi‐photon microscopy of resected tissue sample: (a) collagen (red) and vasa vasorum (green) signals, (b) transparent rendering of collagen and vasa vasorum, (c) segmented vasa vasorum.

The resected tissue sample was stained with CD31 (details in [26]) and scanned with multi‐photon microscopy (MPM) at a slice thickness resolution of approximately 2 μm [26]. As shown in Figure 4a,b, these scans depicted (in 3D) the collagen distribution (red channel) as well as the vasa vasorum (green channel) which runs mainly along the abluminal surface of the tissue sample. The vasa vasorum network was then segmented from these images after applying a partition‐based image calibration strategy that enables precise adjustment of intensity levels in both inter‐slice and intra‐slice directions and then automatically interpolates the intensity threshold levels across partitions to ensure smooth transitions across adjacent partitions, thus facilitating the vasa vasorum segmentation [26] (see Figure 4c).

**FIGURE 5:**
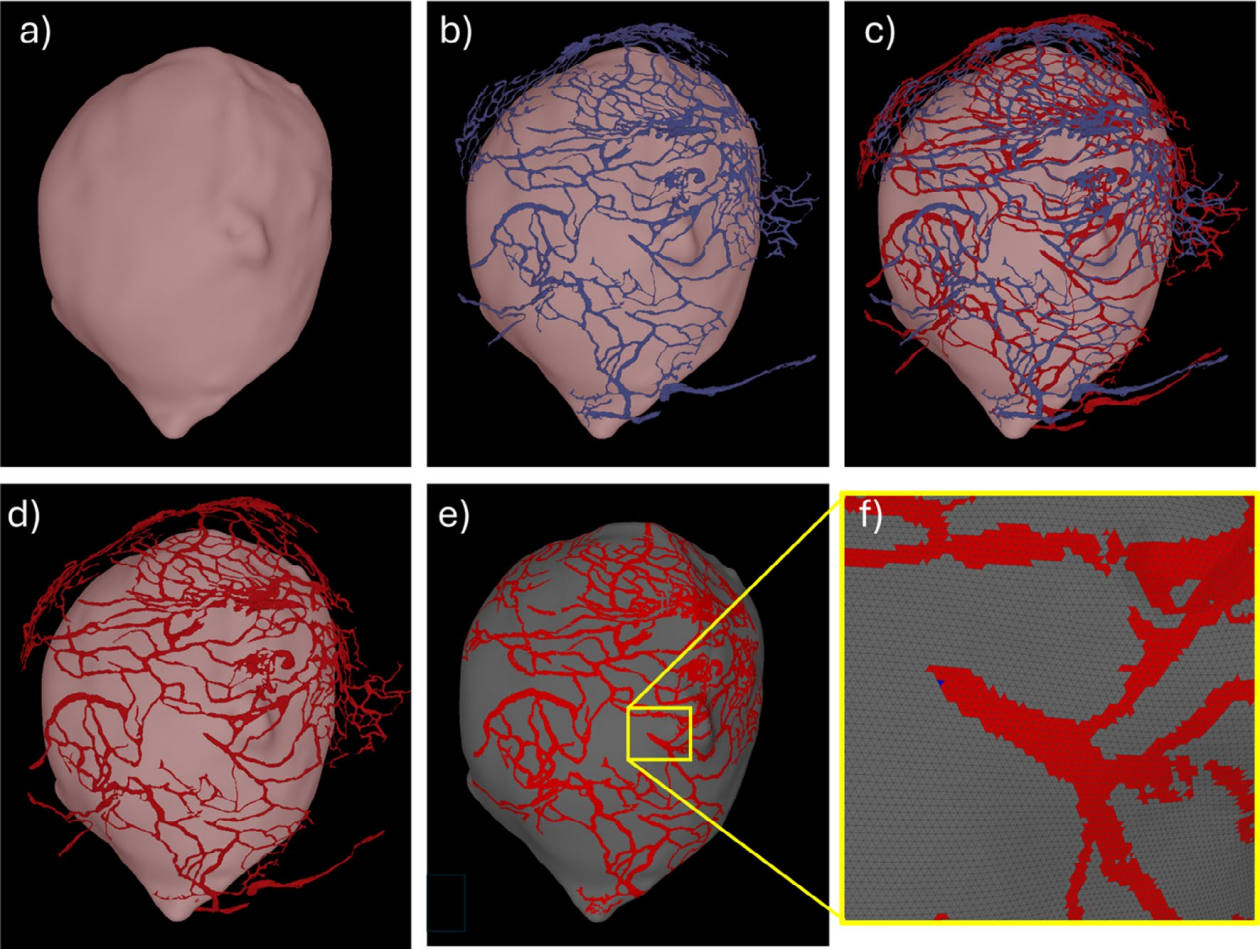
Vasa vasorum modeling: (a) 3D tissue model, (b) aligned vasa vasorum segmentation, (c) wrapping vasa vasorum network, (d) aligned wrapped vasa vasorum network, (e) vasa vasorum projected on tissue model, (f) detail of labeled tissue surface elements.

The 3D reconstruction after segmentation of the vasa vasorum network [25] resulted in a surface triangulation with over 20 million triangles that, even after denoising and smoothing, still contained many topological and geometrical defects, including small holes, disconnected branches, overlapping elements, non‐manifold edges, and so forth. Repairing this surface to render it suitable for subsequent meshing and use in numerical modeling is extremely challenging. As such, a different, simplifying approach was followed in this work, as detailed below and illustrated in Figure 5.

**FIGURE 6:**
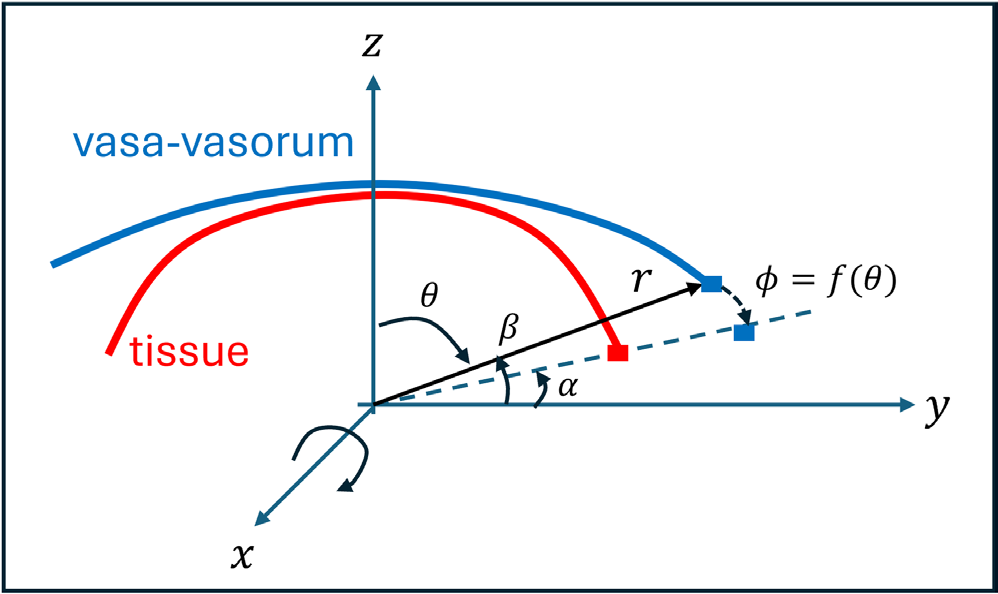
Wrapping the vasa vasorum segmentation (blue) to match the tissue sample (red).

First, the vasa vasorum triangulation was manually registered with the 3D tissue model using previously described tools [27] and the collagen surface as guidance for alignment (see Figure 5a,b). During MPM scanning, the specimen was slightly flattened when imaging under the microscope thus resulting in a vasa vasorum network with a slightly different surface curvature compared to the abluminal surface of the 3D tissue model built from the μCT scan. Therefore, the second step was to wrap the vasa vasorum segmentation to approximately match the curvature of the tissue model reconstructed from the μCT (see Figure 5c, blue = original, red = wrapped). This was achieved by applying a rotation around the *x*‐axis (see Figure 6) given by:

(1) $$R=\left[\begin{array}{ccc} 1 & 0 & 0\\ 0 & \cos\left(\phi \right) & \sin\left(\phi \right)\\ 0 & -\sin\left(\phi \right) & \cos\left(\phi \right) \end{array}\right]$$

where the rotation angle ϕ is computed as a function of the point spherical coordinates as follows:

(2) $$\phi =f\left(\theta \right)=a*\cos\left(b*\left(\theta -\alpha \right)\right)+a$$

with

(3) $$a=\left(\alpha -\beta \right)/2\;\text{and}\;b=\pi /\left(\pi /2-\alpha \right)$$

where α and β the elevations of the boundaries of the tissue and vasa vasorum surfaces as indicated in Figure 6, and the function f is designed to make the surface boundaries coincide while maintaining the regions near the poles (θ=0) roughly unchanged.

The wrapped segmentation aligned to the 3D tissue model is shown in Figure 5d. The last step was then to project the mesh points of the segmentation to the surface of the tissue model and label the corresponding “host” elements. Briefly, for each point of the segmentation, the closest surface element (i.e., host triangle) of the tissue model was found using an octree data structure for efficiently searching in space. The identified host elements were then labeled as belonging to the vasa vasorum. Figure 5e shows the projected vasa vasorum network on the surface of the tissue model, and Figure 5f shows a zoomed view of the labeled surface elements. In this last panel, some of the geometrical details of the vasa vasorum segmentation that make it challenging for use in numerical modeling can be observed.

### 2.6 Oxygen Transport Model

In order to analyze the distribution of oxygen in the aneurysm tissue, a mathematical model was developed that included oxygen diffusion and consumption in the tissue, diffusion from the blood stream across the luminal surface (endothelium), and diffusion from the blood stream along the vasa vasorum across the abluminal surface. The governing equation was as follows:

(4) $$\frac{\partial C}{\partial t}=D\nabla^{2}C+S$$

where C is the oxygen concentration (related to partial pressures through Henry's law C=αP, with α the solubility coefficient), D the oxygen diffusivity in the aneurysm tissue, and S a source. Oxygen consumption in the tissue was modeled using first‐order Michaelis–Menten kinetics as [28]:

(5) $$S=-\frac{M_{0}C}{C_{m}+C}$$

where $M_{0}$ is the baseline oxygen demand, $C_{m}$ is a constant computed as $C_{m}=\alpha_{t}\;P_{0}$ with $\alpha_{t}$ the oxygen solubility in tissue and $P_{0}$ the Michaelis constant for oxygen consumption [28, 29].

Oxygen diffusion from the blood stream across the luminal surface was modeled as a Neumann boundary condition, where the flux (per unit area) across the surface was:

(6) $$q=-P_{l}\;\left(C-C_{b}\right)/\mathrm{ds}$$

where $P_{l}$ represents the permeability of the lumen interface, ds is the distance to the wall, and $C_{b}$ the oxygen concentration in blood, which was assumed to be constant (i.e., differences in oxygen concentration along the flow stream were considered small and neglected) and calculated as follows [30]:

(7) $$C_{b}=C_{0}\frac{P_{b}^{N}}{P_{50}^{N}+P_{b}^{N}}+\alpha_{b}\;P_{b}$$

where $C_{0}$ is the oxygen binding capacity to red blood cells (RBCs), $P_{b}$ is the oxygen partial pressure in flowing blood, $P_{50}$ is the partial pressure at half maximum hemoglobin saturation, N is the Hill equation exponent, and $\alpha_{b}$ is the oxygen solubility in blood.

Similarly, the diffusion from the bloodstream in the vasa vasorum across the abluminal surface of the tissue was modeled as:

(8) $$q=-P_{\mathrm{vv}}\;\left(C-C_{\mathrm{vv}}\right)/\mathrm{ds}$$

where $P_{\mathrm{vv}}$ represents the permeability of the abluminal interface (vasa vasorum), and $C_{\mathrm{vv}}$ the oxygen concentration in blood of the vasa vasorum, which as in previous studies [29, 30], was assumed to be a function of the distance to the vasa vasorum inlet(s):

(9) $$C_{\mathrm{vv}}=C_{b}*\left(1-d_{\text{in}}/d_{\max}\right)$$

where $C_{b}$ is the oxygen concentration in arterial blood, $d_{\text{in}}$ the minimum (geodesic) distance to the inlet(s), and $d_{\max}$ a parameter that modulates oxygen decay along the vasa vasorum.

The governing Equation (4) was numerically solved using an element‐centered finite volumes approach. Integrating the governing equation over an arbitrary control volume V and using the divergence (Gauss) theorem for the first term in the right‐hand side:

(10) $$\int_{V}\frac{\partial C}{\partial t}\;\mathrm{dV}=\underset{A\left(V\right)}{∯}D\nabla C∙n\;\mathrm{dA}+\int_{V}S\;\mathrm{dV}$$

where n is the unit normal along the surface enclosing the volume $A\left(V\right)$. Spatial discretization is achieved by integrating over the volume of each (tetrahedral) element i and assuming the concentration to be constant within the element:

(11) $$V_{i}\frac{\partial C_{i}}{\partial t}=\sum_{j\in N\left(i\right)}D\;A_{\mathrm{ij}}\;\left(C_{i}-C_{j}\right)/ds_{\mathrm{ij}}+V_{i}S_{i}$$

where $V_{i}$ is the element volume, $C_{i}$ the concentration in the element, $S_{i}$ the oxygen consumption per unit volume in the element, the sum is taken over the neighbors across the (triangular) faces of element i, $A_{\mathrm{ij}}$ is the area of the face between elements i and j, and $ds_{\mathrm{ij}}$ is the normal distance between the centroids of elements i and j.

To implement boundary conditions in the luminal or abluminal surfaces, the concentration of the “neighboring” cell j is simply replaced by the $C_{b}$ or $C_{\mathrm{vv}}$ and the corresponding flux multiplied by the corresponding permeability ($P_{l}$ or $P_{\mathrm{vv}}$). The flux across faces not on the luminal or vasa vasorum interface was assumed zero.

The solution of the spatially discretized Equation (11) was advanced explicitly in time using a Runge–Kutta scheme with a timestep selected from the stability condition corresponding to the diffusive and source terms:

(12) $$\Delta t<h_{i}^{2}/2D\;\text{and}\;\Delta t<M_{0}/\left(C_{m}+C_{i}\right)$$

where $h_{i}=\sqrt[{3}]{V_{i}}$ is the element size. Starting with an initial condition corresponding to zero oxygen in the tissue, the solution was advanced until it converged to steady state according to the following condition:

(13) $$\frac{|C_{i}^{n+1}-C_{i}^{n}|}{\left|C_{i}^{n}\right|}<\epsilon$$

where $\epsilon =10^{-5}$ was the convergence tolerance.

The parameters used in the simulations, derived from previous reports [28, 29, 30, 31], are listed in Table 1.

**TABLE 1:** Model parameters used in numerical simulations of oxygen transport across aneurysm tissue.

Parameter	Value	Units	Meaning
N	2.59	—	Hill equation exponent
$P_{0}$	10.5	mmHg	Michaelis constant for $O_{2}$ consumption
$P_{b}$	130	mmHg	$O_{2}$ partial pressure in flowing blood
$P_{50}$	40.2	mmHg	$O_{2}$ partial pressure at half max hemoglobin saturation
$\alpha_{t}$	$3.89\times 10^{-5}$	$\frac{cm^{3}O_{2}}{cm^{3}\;\text{mmHg}}$	$O_{2}$ solubility in tissue
$\alpha_{b}$	$3.1\times 10^{-5}$	$\frac{cm^{3}O_{2}}{cm^{3}\;\text{mmHg}}$	$O_{2}$ solubility in blood
$C_{0}$	0.516	$\frac{cm^{3}O_{2}}{cm^{3}}$	$O_{2}$ binding capacity to RBCs
$M_{0}$	0.02	$\frac{cm^{3}O_{2}}{cm^{3}\;s}$	Baseline $O_{2}$ metabolic demand
D	$2.41\times 10^{-5}$	$cm^{2}/s$	$O_{2}$ diffusivity in tissue
$d_{\max}$	0.4	cm	$O_{2}$ decay along vasa vasorum
$P_{b}$	1.0	—	Luminal permeability
$P_{\mathrm{vv}}$	1.0	—	Abluminal (vasa vasorum) permeability

## 3 Results

### 3.1 Flow and Tissue Characteristics

**FIGURE 7:**
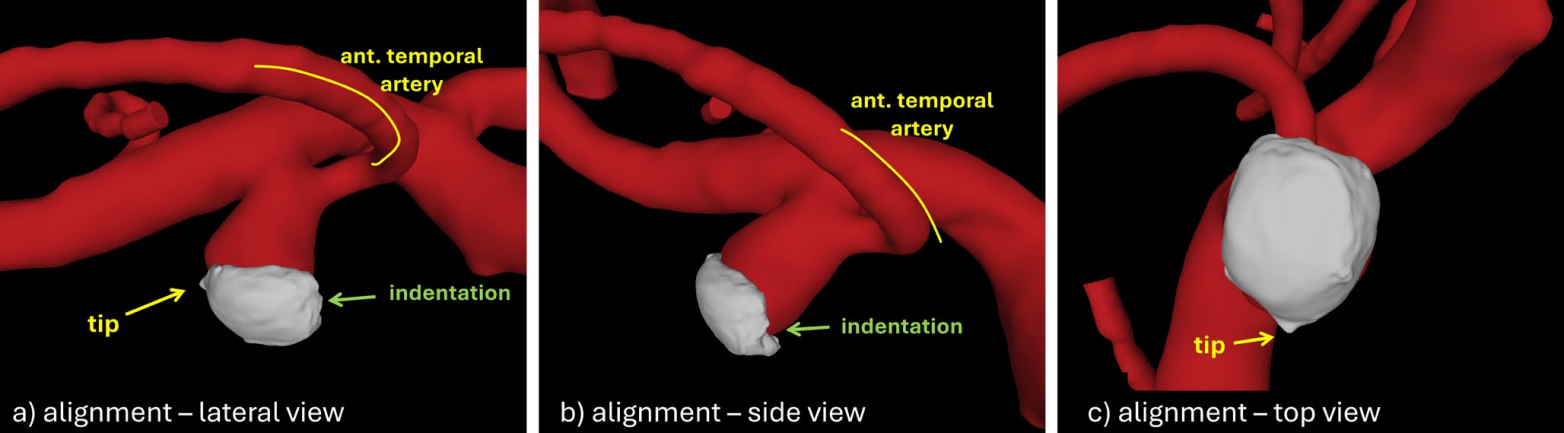
Alignment of tissue model (white) and vascular model (red) from three different viewpoints and highlight of features observed in the surgical video.

The model of the resected tissue sample was aligned to the vascular model in order to inspect and understand the underlying flow conditions. This alignment was carried out manually using tools previously described [27] and under guidance from the surgical videos. For this purpose, the surgical view was first inferred by rotating the vascular tissue to identify structures visible in the video (e.g., the aneurysm, the anterior temporal artery, etc.). Then, the orientation of the resected sample was deduced by observation, in this surgical view, of certain features of the sample such as the indentation left by the first cut and the tip left by the final cut during the harvest. The final alignment is shown in Figure 7 from three viewpoints.

Careful inspection of the surgical video reveals that the aneurysm wall looks quite thick with a whitish appearance, except near the neck close to the origin of the anterior temporal artery where there is a small reddish region which is typically associated with thinner walls [4] (see Figure 2a). During the resection of the sample as it is being cut it is also possible to observe that the walls of the specimen also look quite thick (see Figure 2d,e). Indeed, the wall thickness determined from the ex vivo μCT analysis ranged from approximately 200–733 μm (see Figure 3c). The thinner part of the sample was towards the “tip” while the thicker part towards the “indentation” and to the right side of this figure.

**FIGURE 8:**
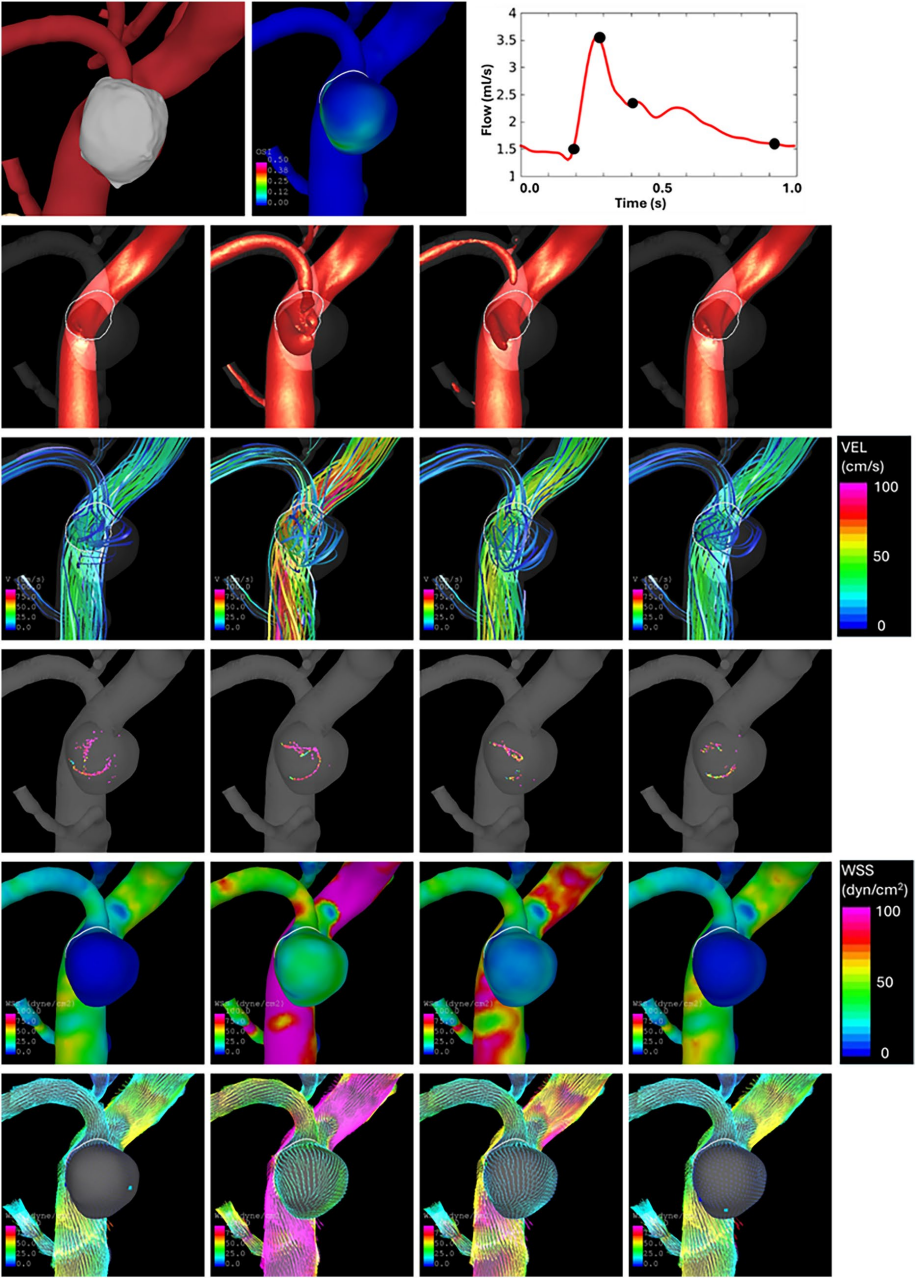
Flow visualization from top view (top‐left panel) at four instants of time (top‐right panel). Top to bottom rows: Oscillatory shear index, inflow jet, flow streamlines, vortex core lines, wall shear stress magnitude, wall shear stress vectors, and critical points.

**FIGURE 9:**
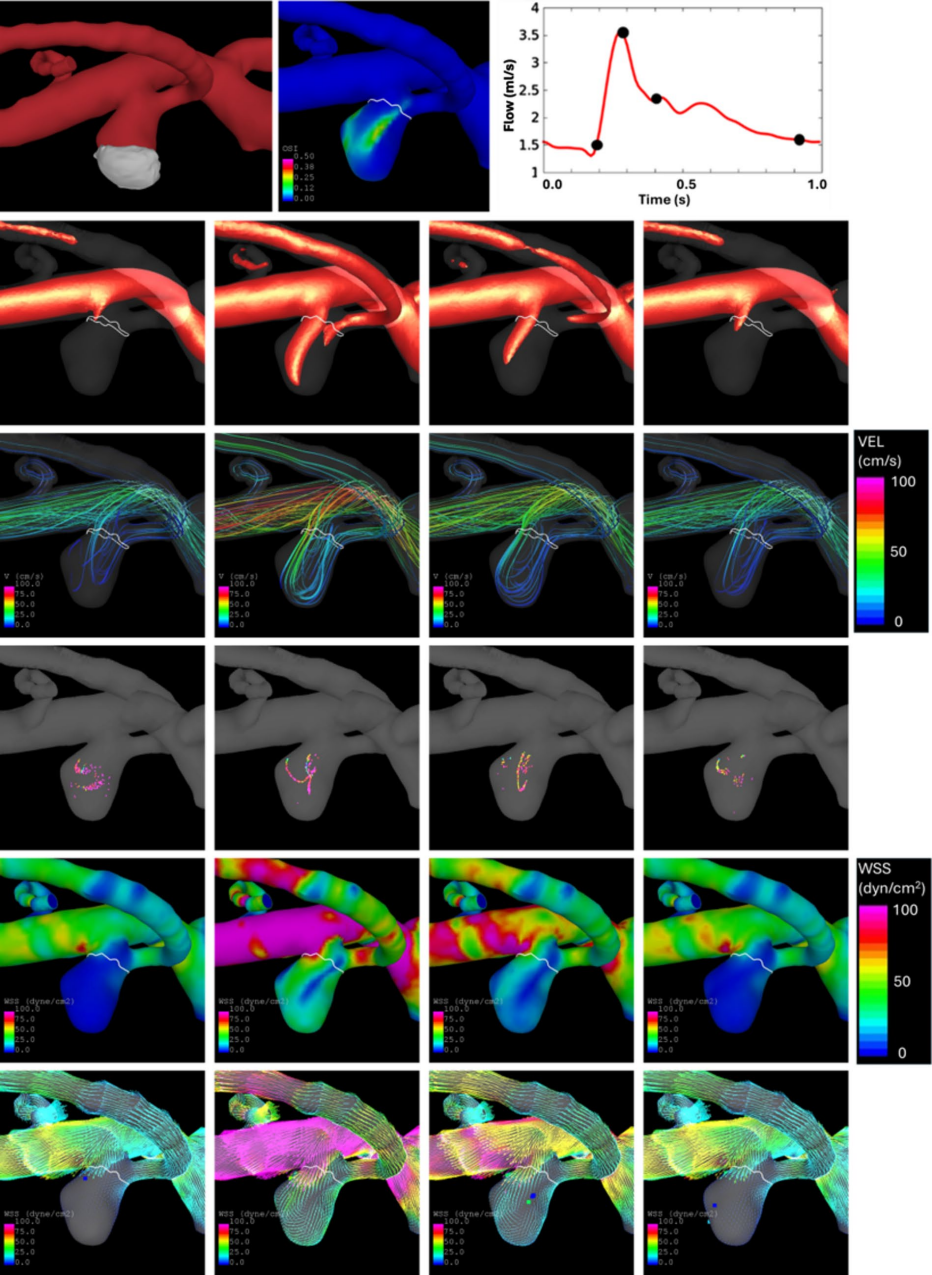
Flow visualization from side view (top‐left panel) at four instants of time (top‐right panel). Top to bottom rows: Oscillatory shear index, inflow jet, flow streamlines, vortex core lines, wall shear stress magnitude, wall shear stress vectors, and critical points.

Blood flow visualizations from the top view and the side view are presented in Figures 8 and 9, respectively. The first row of these figures shows the orientation, the distribution of oscillatory shear index (OSI), and the four instants of time during the cardiac cycle corresponding to the subsequent visualizations. The second row shows the inflow jet rendered as a high‐speed iso‐velocity surface. The third row shows the intra‐aneurysmal flow pattern through instantaneous streamlines. The fourth row shows vortex centerlines. The fifth and sixth rows show the magnitude of wall shear stress (WSS) along with the WSS vectors and critical points.

Overall, the entire aneurysm is exposed to low WSS and a single vortex flow pattern. The thinner part of the sample (towards the tip) seems to be exposed to slightly higher WSS and faster flow, while the thicker part (towards the indentation) seems to be exposed to lower WSS and slower flow. Additionally, a relatively strong WSS gradient (and a saddle critical point) is observed, especially during systole, on the side of the aneurysm towards the origin of the ATA (see Figure 9, row 6) where thinner walls were observed in the surgical video.

These results highlight the hemodynamic conditions that promote local aneurysm wall thickening at the dome from where the tissue sample was collected, namely slow vortical flow with low WSS.

### 3.2 Oxygen Distribution Without Vasa Vasorum

The first oxygen transport simulation was carried out without considering oxygen diffusion from the vasa vasorum to the tissue. For this purpose, the permeability of the vasa vasorum/tissue interface was set to zero: $P_{\mathrm{vv}}=0$, and oxygen was only allowed to diffuse across the luminal interface. The transport simulation converged to $\epsilon =10^{-5}$ in approximately 128 K explicit steps with a timestep size of $\Delta t=7.8\times 10^{-5}\;s$ and took approximately 21 h running on 32 cores in shared memory mode.

**FIGURE 10:**
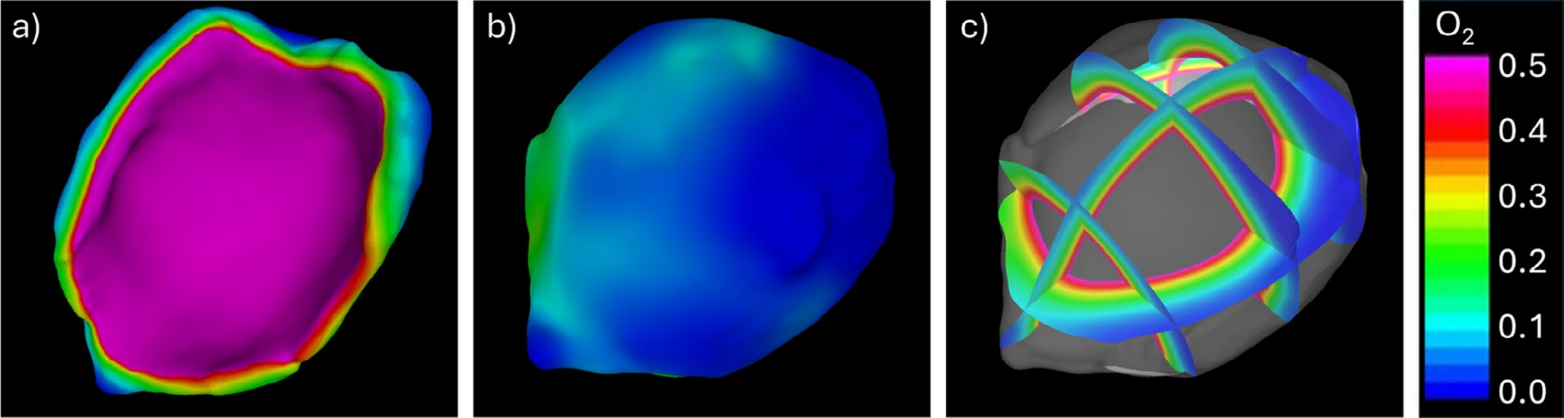
Distribution of oxygen concentration obtained without diffusion from the vasa vasorum: (a) luminal surface, (b) abluminal surface, (c) distribution on cutting planes across the thickness.

Visualizations of the steady state oxygen concentration in the absence of vasa vasorum are presented in Figure 10. These results indicate that in the thicker parts of the aneurysm sample the oxygen concentration towards the abluminal side becomes quite low, with a concentration of almost zero, most likely inducing hypoxia. This inability of the oxygen reaching the abluminal side of the wall is caused by the oxygen consumption within the thickened aneurysm wall. Note that, with the model parameters used in these simulations, in regions where the wall thickness is comparable with a normal MCA artery (approximately 200 μm) the oxygen distribution reaches a more favorable concentration (approximately 0.3).

### 3.3 Oxygen Distribution With Vasa Vasorum

**FIGURE 11:**
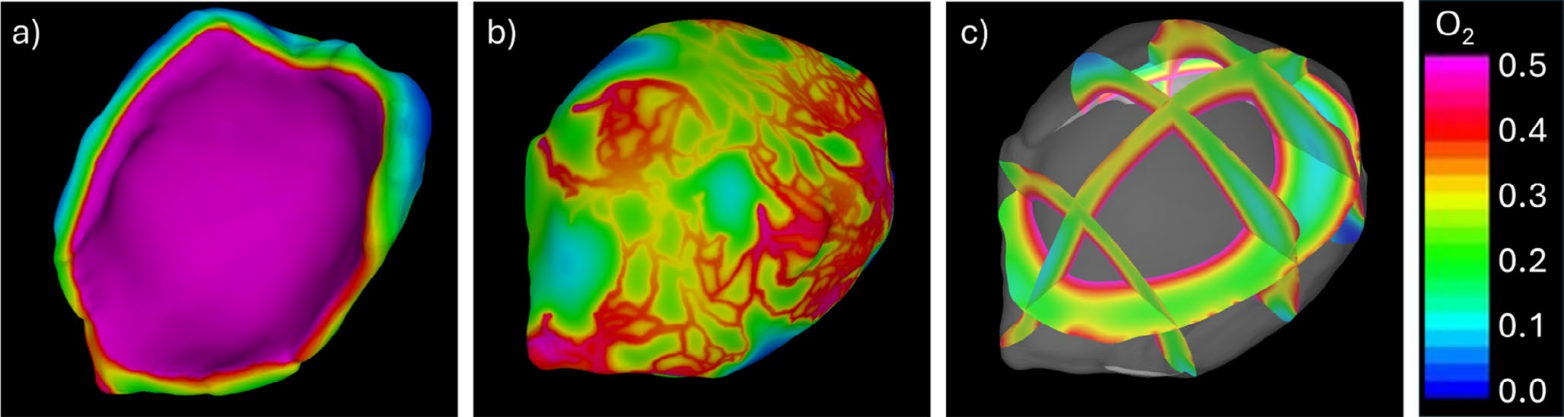
Distribution of oxygen concentration obtained with model that included diffusion from the vasa vasorum: (a) luminal surface, (b) abluminal surface, (c) distribution on cutting planes across the thickness.

The second oxygen transport simulation included the diffusion from the vasa vasorum on the abluminal side. In this case, the vasa vasorum permeability was set to the same value as the one on the lumen side. The corresponding results are presented in Figure 11. In this case, the oxygen distribution resembles that of a normal artery everywhere, including the thick wall regions that contain a denser vasa vasorum network and the thinner wall regions that contain fewer or no vasa vasorum vessels. Thus, the presence of the vasa vasorum seems to prevent hypoxic conditions within the thickened aneurysm wall.

## 4 Discussion

Previous models of oxygen transport in vascular walls with vasa vasorum focused mainly on idealized atherosclerotic plaques or abdominal aortic aneurysms [32, 33], but did not consider the geometry and topology of the vasa vasorum network or the actual geometry of the vessel and its wall thickness. Similarly, models of oxygen transport in IAs have included patient‐specific vascular geometries but not the vessel wall thickness or the vasa vasorum network [34]. The current study investigated the oxygen transport in a cerebral aneurysm tissue sample, including the wall thickness and vasa vasorum network extracted from high‐resolution ex vivo images.

Flow analysis of the aneurysm investigated in this study revealed that most of the aneurysm is exposed to a hemodynamic environment characterized by low WSS, which previous studies have identified as favorable for atherosclerotic remodeling, wall thickening, and aneurysm growth [4, 5, 35].

In our previous analysis [26], the vasa vasorum was observed to form a plexus or network of small vessels, which ran along the adventitial side of the aneurysm wall, with only a few branches penetrating and terminating inside the wall. Furthermore, a local association was found between increased wall thickness and increased density of the vasa vasorum network.

In the current study, results first obtained without including oxygen diffusion from the vasa vasorum network indicate that if oxygen only diffuses from the blood stream across the luminal surface, its concentration in tissue regions with very thick walls (above 600 μm) decays to nearly zero towards the abluminal surface. In other words, the oxygen supply from the luminal blood stream does not seem sufficient to sustain the oxygen consumption by mural cells and reach the adventitial side of thick walls, most likely creating local hypoxic conditions. On the other hand, in regions where the wall had a normal thickness (i.e., around 200 μm) the oxygen concentration on the adventitial side reached suitable levels. Notably, these results were obtained with typical model parameter values derived from previous reports [28, 29, 30, 31].

Secondly, including additional oxygen diffusion from the vasa vasorum network on the adventitial side produced an oxygen distribution in regions of thick walls with concentration values resembling those obtained without vasa vasorum in regions of normal thickness. In other words, the vasa vasorum supplied the extra oxygen needed to regularize the oxygen concentration throughout the aneurysm wall, especially in regions of enlarged wall thickness and denser vasa vasorum.

These findings provide support to the idea that the vasa vasorum network develops as the aneurysm wall thickens in response to low flow conditions that favor cell proliferation, inflammation, and atherosclerotic changes. Presumably, as the aneurysm wall thickens, the oxygen demand increases and induces angiogenesis through expression of hypoxia factors. Thus, the development of the vasa vasorum provides a more robust oxygenation of the wall and enables further thickening and remodeling of the wall and enlargement of the aneurysm. Impairment of this mechanism may predispose the aneurysm wall to weakening or maladaptive remodeling and ultimately failure and aneurysm rupture. This conjecture needs to be further explored, for example, by studying the expression of hypoxia markers and their relationship with the vasa vasorum network and oxygen distribution. Furthermore, alternative promoters of vasa vasorum development, such as infiltration and action of inflammatory cells and atherosclerotic restructuring of the aneurysm wall, need to be simultaneously studied along with the wall oxygenation.

The current study has several limitations: (1) Oxygen consumption was assumed uniform throughout the aneurysm wall. The model could be improved by incorporating cell density distributions derived from ex vivo measurements. (2) Oxygen concentration on the luminal side was assumed constant, which is a reasonable approximation given the small size of the tissue sample. The model could be improved by coupling it to oxygen transport simulations along the blood stream (simulated by CFD). (3) Oxygen transport along the vasa vasorum network was simplified with a distance to the inlets function (as in previous studies reported in the literature). The model could be improved by incorporating convective transport along the vasa vasorum network, but this would require repairing the vasa vasorum network reconstruction to render it suitable for flow and transport simulations. (4) Alignment of the vasa vasorum and tissue models reconstructed from independent ex vivo images was carried out manually by visual inspection, co‐registering visible surface features. This process could be improved by adding physical markers (fiducials) to the tissue sample that could be used to register the corresponding segmentations. Furthermore, the vasa vasorum network was largely within 50 μm of the adventitial network and therefore, for simplicity it was modeled as lying on the surface. (5) The study focused on a single aneurysm sample with thickened walls, which is not likely to represent the full range of wall characteristics of the entire aneurysm population. Further studies with multiple cases and different wall characteristics are needed to generalize the preliminary findings reported here and better understand the prevalence and development of vasa vasorum networks and their function.

Despite these limitations, the simple (but not trivial) computational model (along with its parameter values) presented in this study produced compelling results that could logically explain the observed association between aneurysm wall thickness and the presence of a dense vasa vasorum network.

## 5 Conclusions

Numerical simulations of the oxygen transport across the walls of an IA demonstrate that the vasa vasorum externa observed under multi‐photon microscopy provided adequate oxygen supply to the outer layers of the wall in regions that underwent significant wall thickening in response to low flow conditions. Without the vasa vasorum, due to consumption throughout the wall, the oxygen demand in the outer layers of thickened walls cannot be met by the oxygen diffusion from the luminal surface. Thus, these models support the idea that local hypoxic conditions in regions of increased wall thickness stimulate the development of the vasa vasorum network on the adventitial surface.

## Ethics Statement

The protocol for patient consent, handling of patient data, tissue harvest, and analysis was approved by the institutional review boards at Allegheny General Hospital, the University of Pittsburgh, and George Mason University.

## Conflicts of Interest

The authors declare no conflicts of interest.

## Data Availability

The models used in this study can be made available to interested researchers upon request.
